# Mitochondrial Bioenergetics in Different Pathophysiological Conditions 4.0

**DOI:** 10.3390/ijms26073396

**Published:** 2025-04-05

**Authors:** Daniela Valenti, Anna Atlante

**Affiliations:** Institute of Biomembranes, Bioenergetics and Molecular Biotechnologies (IBIOM)-CNR, Via G. Amendola122/O, 70126 Bari, Italy; a.atlante@ibiom.cnr.it

Mitochondria are complex and multifunctional intracellular organelles involved in many aspects of cellular life, with a primary role in energy production via oxidative phosphorylation process. Mitochondria constitute a dynamic network of signaling platforms, playing several key roles in cell metabolism, proliferation, and survival [1]. Dysfunctional mitochondria are critically involved in the pathogenesis of a wide range of metabolic, neurodegenerative, immune, and neoplastic disorders [2].

In the fourth volume of this Special Issue, four review papers and two original articles were published, dealing with compelling topics related to mitochondrial bioenergetics in certain pathophysiological contexts.

Alzheimer’s disease (AD) is the most common neurodegenerative disorder, characterized by a progressive failure in cognitive function due to the progressive loss of neurons in the forebrain and other brain areas [3]. Mitochondrial dysfunction has been established as an early and prominent feature of the disease [4]. Mitochondrial changes in AD have also been shown to correlate with reduced energy metabolism and oxidative stress. In their review, García-Bermúdez et al. focused on the compelling parallels between the retinal and cerebral manifestations of AD, providing a detailed account of the retinal structure and cell alterations observed in the context of AD [5]. The review delves into the intricate interplay between retinal cells and the resulting bioenergetic deterioration, oxidative stress, and mitochondrial dysfunction, discussing the promising diagnostic techniques already available for the detection of retinal and ocular alterations in AD, which offer the dual advantage of early diagnosis and the potentially dynamic monitoring of disease progression [5].

Mitochondrial dysfunction is widely recognized as a common central pathway involved in the pathogenetic processes of Parkinson’s disease (PD), the second most common neurodegenerative disorder, characterized by the selective loss of dopaminergic neurons of the substantia nigra [6]. The interesting data shown in the study by Mironova et al. highlight the therapeutic potential of the natural metabolite uridine in preventing and slowing the development of PD. The mechanism of the neuroprotective action of uridine may be related, among other aspects, to its ability to activate the mitochondrial channel mitoKATP, and prevent the progression of mitochondrial dysfunction in brain tissue [7].

A growing body of evidence points to mitochondria as key targets for the development of therapeutics aimed at resolving both metabolic and vascular dysfunction. MitoNEET was identified as a binding target for a class of antidiabetic drugs and is now recognized for its role in regulating various crucial cellular processes. Indeed, mitoNEET has demonstrated promising potential as a therapeutic target in various chronic diseases, encompassing cardiovascular and metabolic diseases. In their paper, Tam and Sweeny show an overview of the molecular mechanisms of mitoNEET, with an emphasis on their implications for cardiometabolic diseases [8].

Mitochondria are key organelles in crucial sperm functions, such as motility, hyperactivation, capacitation and fertilization, with sperm mitochondria being the driving force behind human spermatozoa activities (for a narrative review, see [9]).

In their study, Ferigolo et al. evaluated the effects of the isoflavone genistein as a mitochondrial modulator on the quality of the spermatozoa of Wistar rats, focusing on mitochondrial function [10]. Genistein treatment was found to enhance the overall mitochondrial oxygen consumption, increasing plasmatic testosterone levels by showing pro-spermatogenesis action with a better mitochondrial profile, and without systemic toxicity. Short-term treatment with a relevant dose of genistein resulted to be effective in improving sperm mitochondrial efficiency.

Mitochondrial dysfunction is involved in periodontitis, defined as a chronic inflammatory disease caused by *Porphyromonas gingivalis* that leads to the destruction of periodontal tissues, the resorption of alveolar bone, and even the loosening and loss of teeth. The periodontal inflammatory response results in the abnormal release of mitochondrial contents. In their paper, Luo et al. reviewed the emerging involvement of mitochondria in the pathogenesis of periodontitis, which results in mitochondrial metabolic transformations and induces an imbalance in mitochondrial quality control. This leads to the accumulation of defective mitochondria and an increase in mitochondrial ROS sustained by an efflux of damage-associated molecular patterns that trigger inflammatory responses [11]. The mitochondrial pathway was found to be inextricably linked to the pathology of periodontitis caused by *P. gingivalis*.

Mitochondria are dynamic, energy-transforming, biosynthetic, and signaling organelles that actively transduce biological information. Novel research has shown that the mitochondrial function of mammalian cells can be modulated by various energetic stimuli, including sound vibrations. Regarding acoustic vibrations, in recent studies, the effects of different sound stimuli and musical styles on cellular function and mitochondrial activity were evaluated and compared in human cells cultured in vitro to investigate the underlying responsible molecular mechanisms [12]. In their narrative review, Valenti and Atlante undertook a multilevel path from the macro to the intracellular microcosm, discussing the intimate vibrational activities of sound that permeate and shape living matter, and the impact of sound on biological systems, focusing their discussion primarily on recent evidence showing the competence of mitochondria to act as energetic portals capable of sensing and transducing the subtle informational biofields of sound vibrations [13].

Mitochondria could participate in the “symphony of cellular oscillatory patterns”, acting as dynamic bio-informational “trackers”, transducing and promoting molecular and informational transfer in and out of the organelles and across the cell on the nuclear level, and enabling the regulation of the expression of transcriptional regulators and transcription factors [14].

## References

[1] Lisowski P., Kannan P., Mlody B., Prigione A. (2018). Mitochondria and the dynamic control of stem cell homeostasis. EMBO Rep..

[2] Wallace D.C. (2005). A Mitochondrial Paradigm of Metabolic and Degenerative Diseases, Aging, and Cancer: A Dawn for Evolutionary Medicine. Annu. Rev. Genet..

[3] Swerdlow R.H. (2018). Mitochondria and Mitochondrial Cascades in Alzheimer’s Disease. J. Alzheimer’s Dis..

[4] Nitzan K., Benhamron S., Valitsky M., Kesner E.E., Lichtenstein M., Ben-Zvi A., Ella E., Segalstein Y., Saada A., Lorberboum Galski H. (2019). Mitochondrial Transfer Ameliorates Cognitive Deficits, Neuronal Loss, and Gliosis in Alzheimer’s Disease Mice. J. Alzheimer’s Dis..

[5] García-Bermúdez M.Y., Vohra R., Freude K., Wijngaarden P.V., Martin K., Thomsen M.S., Aldana B.I., Kolko M. (2023). Potential Retinal Biomarkers in Alzheimer’s Disease. Int. J. Mol. Sci..

[6] Marino B.L., De Souza L.R., Sousa K.P., Ferreira J.V., Padilha E.C., Da Silva C.H., Taft C.A., Hage-Melim L.I. (2020). Parkinson’s Disease: A Review from Pathophysiology to Treatment. Mini-Rev. Med. Chem..

[7] Mironova G.D., Mosentsov A.A., Mironov V.V., Medvedeva V.P., Khunderyakova N.V., Pavlik L.L., Mikheeva I.B., Shigaeva M.I., Agafonov A.V., Khmil N.V. (2024). The Protective Effect of Uridine in a Rotenone-Induced Model of Parkinson’s Disease: The Role of the Mitochondrial ATP-Dependent Potassium Channel. Int. J. Mol. Sci..

[8] Tam E., Sweeney G. (2023). MitoNEET Provides Cardioprotection via Reducing Oxidative Damage and Conserving Mitochondrial Function. Int. J. Mol. Sci..

[9] Kumar N. (2023). Sperm Mitochondria, the Driving Force behind Human Spermatozoa Activities: Its Functions and Dysfunctions—A Narrative Review. Curr. Mol. Med..

[10] Ferigolo M., Nardi J., Freddo N., Ferramosca A., Zara V., Dallegrave E., Macedo M.B., Eller S., de Oliveira A.P., Biazus I.C. (2023). Evaluation of Genistein as a Mitochondrial Modulator and Its Effects on Sperm Quality. Int. J. Mol. Sci..

[11] Luo S., Xu T., Zheng Q., Jiang A., Zhao J., Ying Y., Liu N., Pan Y., Zhang D. (2024). Mitochondria: An Emerging Unavoidable Link in the Pathogenesis of Periodontitis Caused by *Porphyromonas gingivalis*. Int. J. Mol. Sci..

[12] Feng Q., Wang L., Chen Y., Teng J., Li M., Cai Z., Niu X., Rein G., Yang Q., Shao X. (2022). Effects of different music on HEK293T cell growth and mitochondrial functions. Explore.

[13] Valenti D., Atlante A. (2024). Sound Matrix Shaping of Living Matter: From Macrosystems to Cell Microenvironment, Where Mitochondria Act as Energy Portals in Detecting and Processing Sound Vibrations. Int. J. Mol. Sci..

[14] Muehsam D., Ventura C. (2014). Life rhythm as a symphony of oscillatory patterns: Electromagnetic energy and sound vibration modulates gene expression for biological signalling and healing. Glob. Adv. Health Med..

